# How mRNA therapeutics are entering the monoclonal antibody field

**DOI:** 10.1186/s12967-019-1804-8

**Published:** 2019-02-22

**Authors:** Lien Van Hoecke, Kenny Roose

**Affiliations:** 1 VIB Center for Medical Biotechnology, VIB, Ghent, Belgium; 2 Department of Biomedical Molecular Biology, Ghent University, Ghent, Belgium; 3 Departement of Biochemistry and Microbiology, Ghent University, Ghent, Belgium

**Keywords:** mRNA, mRNA therapeutic, Antibody therapy, Passive immunization, mRNA design, mRNA technology

## Abstract

In 1975, Milstein and Köhler revolutionized the medical world with the development of the hybridoma technique to produce monoclonal antibodies. Since then, monoclonal antibodies have entered almost every branch of biomedical research. Antibodies are now used as frontline therapeutics in highly divergent indications, ranging from autoimmune disease over allergic asthma to cancer. Wider accessibility and implementation of antibody-based therapeutics is however hindered by manufacturing challenges and high development costs inherent to protein-based drugs. For these reasons, alternative ways are being pursued to produce and deliver antibodies more cost-effectively without hampering safety. Over the past decade, messenger RNA (mRNA) based drugs have emerged as a highly appealing new class of biologics that can be used to encode any protein of interest directly in vivo. Whereas current clinical efforts to use mRNA as a drug are mainly situated at the level of prophylactic and therapeutic vaccination, three recent preclinical studies have addressed the feasibility of using mRNA to encode therapeutic antibodies directly in vivo. Here, we highlight the potential of mRNA-based approaches to solve several of the issues associated with antibodies produced and delivered in protein format. Nonetheless, we also identify key hurdles that mRNA-based approaches still need to take to fulfill this potential and ultimately replace the current protein antibody format.

## Antibodies: from a natural defense mechanism to a frontline therapeutic

### The genesis of antibody therapeutics: the protein-format

The foundation of the antibody industry was laid in 1975, when Köhler and Milstein developed the hybridoma technology. This technique made the production of an unlimited amount of identical or monoclonal antibodies (mAbs) possible [1]. It also led to the assignment of the Nobel prize for Medicine and Physiology in 1984 and to the license of the first mAb therapeutical in 1986, namely Orthoclone OKT3 (muromonab-CD3) to treat graft-versus-host disease [2].

**Fig. 1:**
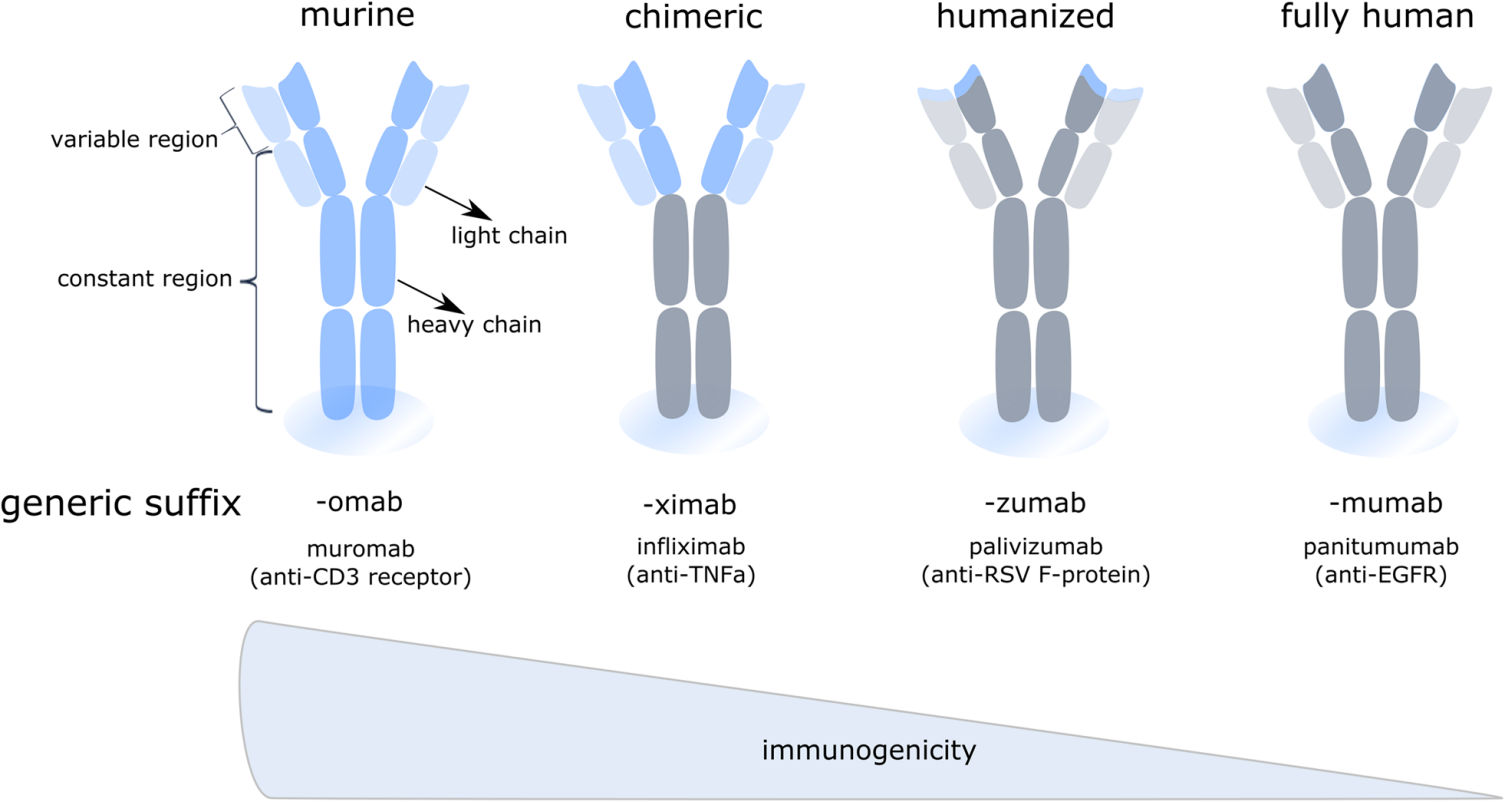
Overview of monoclonal antibody variants used in therapy. Next to classical fully murine (left) or human monoclonal antibodies (right), recombinant species are used in therapy (middle). These include chimeric mAbs, composed of human constant regions and murine variable regions, and humanized mAbs, where the hypervariable CDR-domains of the murine antibody are grafted on a human antibody. Clinically applied examples of each are given, including their targets between brackets

Despite initial excitement, it soon became clear that first generation mAbs were facing serious problems with immunogenicity provoked by the murine origin of these mAbs. Fortunately, in the early 90s, molecular biology and recombinant antibody production technology in combination with detailed descriptions of antibody gene coding, induced a revolution in the mAbs industry. These new technologies indeed paved the way for the generation of improved recombinant mAb formats (Fig. 1), that gradually contained less murine sequences and ultimately culminated in the design of fully human antibodies [3].

Not surprisingly, the clinical use of mAbs today represents a rapidly growing billion dollar market for the biopharmaceutical industry, with projected combined worldwide sales of nearly $125 billion in 2020 [4]. mAb therapies are now available to treat disorders ranging from rheumatoid arthritis that affects millions of patients to rare diseases with just a few thousand patients like mantle cell lymphoma [4]. Currently, there are 76 mAb approved by the European Medicines Agency (EMA) and/or the US Food and Drug Administration (FDA) for therapeutic use [5] and over 50 mAb are being investigated in late-stage clinical studies [6]. Approximately six new mAb products are being licensed every year [5]. A striking example where the therapeutic use of mAb has revolutionized the treatment options for patients is the development of so called check point inhibitor mAb that boosted the cancer immunotherapy field. Check point inhibitors are now one of the most successful and important strategies for treating cancer patients.

Notwithstanding the monoclonal antibody industry is one of the fastest growing pharmaceutical industries, the technical, regulatory, and strategic Chemistry, Manufacturing, and Controls (CMC) activities necessary to successfully advance new monoclonal antibody products to clinical trials and to market approval are huge. These challenges are inherent to the current manufacturing process of mAbs as the production of mAbs is performed in mammalian cell lines followed by purification from complex media, implying that an extensive purification process is needed to obtain a safe formulatable antibody from the cell culture supernatant free from viruses and other contaminants. In addition, monoclonal antibodies are prone to a wide variety of post-translational modifications, including glycosylation, deamidation, oxidation, incomplete disulfide bond formation, N-terminal glutamine cyclization, and C-terminal lysine processing. As these modifications can strongly impact the biological activity and therapeutic properties of the mAbs, they need to be characterized and controlled, necessitating the costly development and implementation of numerous analytical tools to assess these Quality Attributes. All these aspects lead to a challenging production process. At the same time, regulatory agencies ask for enhanced quality while health care systems demand lower process costs.

### The body as its own bioreactor: the nucleic-acid-format

An elegant solution to circumvent the problems of complex production and purification processes and aberrant posttranslational modifications of the antibody, is to deliver the genetic information of the antibody itself. Transient gene transfer aims at administering the mAb-encoding nucleotide sequences in DNA or mRNA form, rather than the mAb protein itself, directly to patients. This allows for the in situ production of biologicals in a cost- and labor-effective manner, potentially for a prolonged period of time.

As proteins are composed of 20 different amino acids as building blocks, the physicochemical properties differ from protein to protein, implying that for each protein the buffer for storage and formulation should be optimized specifically. DNA and mRNA on the other hand are composed of a mere four building blocks, i.e. the four nucleosides adenosine, guanosine, uridine and cytidine. The overall structure is a sugar-phosphate backbone nucleic acid polymer with a strong negative charge which, importantly, has largely consistent physicochemical characteristics regardless of the protein sequence it encodes. As a direct consequence, the production and purification process does not need to be tailored for each and every individual DNA or mRNA encoding antibody drug product. Another desirable characteristic of nucleic acid encoding antibodies is the fact that in case of an outbreak of a disease or infective strain, the sequence for the antibody can be designed very rapidly and produced in high quantities without the need for specific optimization of these processes [7, 8].

The field of DNA-based therapeutics sparked in 1990, when Wolff et al. showed that injection of naked plasmid DNA (pDNA) in the quadriceps of mice resulted in the local expression of the encoded protein [9]. Different preclinical studies have shown that the delivery of DNA-encoded antibodies is able to protect against different infectious diseases, like dengue virus, respiratory syncytial virus and chikungunya virus [10–12]. Currently, several of these pDNA-encoded antibody designs are being evaluated in phase II–III clinical trials. Nonetheless, a pDNA-based pharmaceutical for humans has not been marketed so far. This can be explained by the concerns surrounding potential integration of the pDNA into the host genome and the fear of anti-DNA autoantibodies. However, it has been found that these effects are minimal, although integration must be monitored for each DNA-encoded antigen separately [13, 14]. Next to these concerns, the vaccination site plays a crucial role in the efficacy of DNA vaccines. The majority of clinical trials use intramuscular (IM) injection. But the efficacy of IM DNA vaccination depends on the injection volume, as the volume causes an increased local pressure that enhance cell uptake and slightly induce tissue damage. This tissue damage in its turn encourages the recruitment and maturation of antigen presenting cells (APCs). As a consequence, IM injections in rodents are likely to result in a more robust immune response than in a larger animals and human patients [15, 16].

Although research has been mainly focused on the development of pDNA, the limitations associated with these ‘classical’ approaches and the recent improvements in stability and translatability of in vitro transcribed (IVT) mRNA, have recently led to an increased interest in mRNA as a delivery vector. In addition to safer pharmaceutical properties, such as no risk of genome integration, the transient expression of mRNA-encoded antibodies enables a more controlled exposure, with more protein production during peak expression compared to naked pDNA [17]. Because additional sequences such as plasmid backbone are lacking in mRNA vaccines, the pre-existence or induction of anti-vector antibodies is of no issue. Moreover, mRNA is produced by an in vitro transcription process without any mammalian cells, so there is no risk for adventitious viruses. Also with the use of mRNA, the coding information of the antibody is delivered to the cytoplasm and is directly in vivo translated to the encoded protein. In this way, risks of aberrant posttranslational modifications, inherent to the protein delivery platform, are circumvented. From a regulatory point of view, mRNA administration has recently been classified by the EMA as an Advanced Therapy Medicinal Product (ATMP), and more precise as a Gene Therapy Medicinal Product (GTMP).

All the advantages mentioned above highlight the tremendous potential of IVT mRNA to reshape antibody mediated therapies. Nonetheless, to successfully replace the current protein antibody format, mRNA approaches will need to surpass their own challenges, which are mainly situated at the level of delivery and of immunogenicity.

## mRNA as new appealing therapeutic platform

### Synthetic mRNA as an attractive chemical blueprint

Several years ago, Wolff et al. and others showed that IVT mRNA is translated into the encoded protein after transfection [9, 18–20], using the protein synthesis machinery of the transfected cell itself [21, 22]. Despite this promising discovery, at that time mRNA was considered as particularly unstable as under normal conditions, unmodified IVT mRNA is degraded by the omnipresent extra- or intracellular ribonucleases. This unstable nature of mRNA made its therapeutic use a challenging idea. Fueled by this challenge and the promising opportunities of mRNA, researchers investigated a series of modifications to the vector used to produce mRNA as well as to the synthetic mRNA itself in order to improve the molecule stability and protein translation [23–25].

**Fig. 2:**
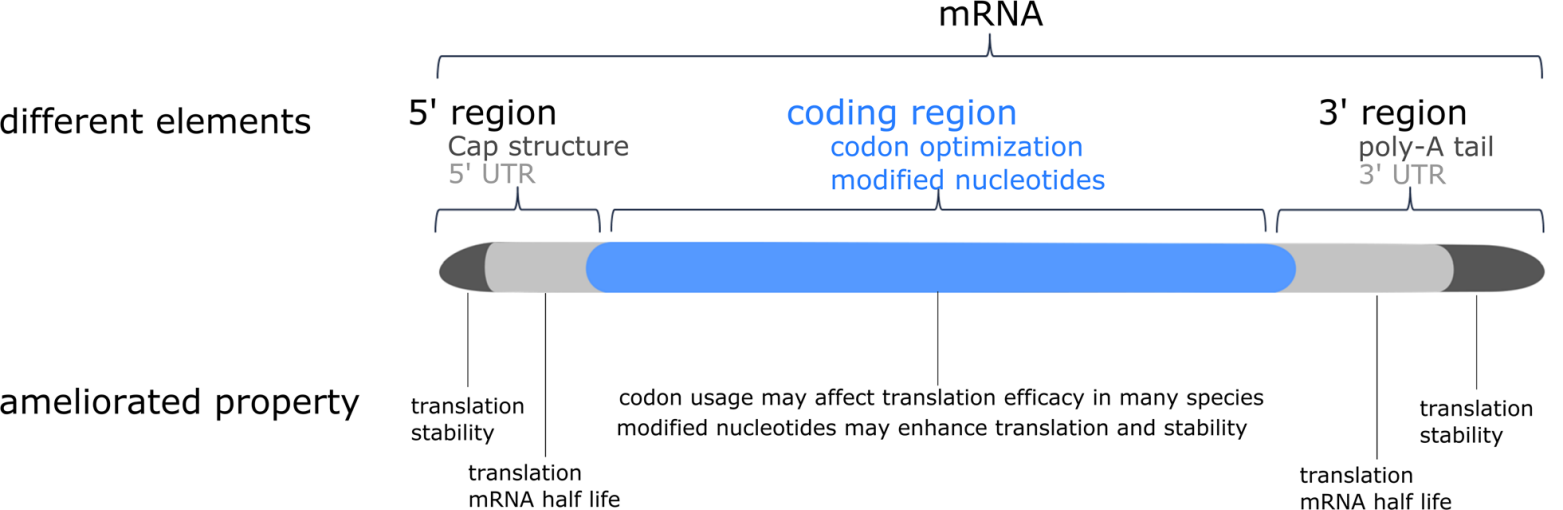
Schematic representation of optimized mRNA. The mRNA consists of different in cis-acting elements from 5′ to 3′: cap structure, 5′UTR, coding region with modified nucleotides, 3′ UTR and a poly-A tail

The template for in vitro transcription of mRNA consists of five in cis-acting structural elements, namely from 5′ to 3′ end: (i) the optimized cap structure, (ii) the optimized 5′ untranslated region (UTR), (iii) the codon optimized coding sequence, (iv) the optimized 3′ UTR and (v) a stretch of repeated adenine nucleotides (polyA tail) [26]. These cis-acting structural elements are constantly further optimized in the endeavor for better mRNA features. Figure 2 is a schematic representation of optimized mRNA. The poly-A-tail and the cap structure are important for the efficient translation of the mRNA and to stabilize the mRNA against decay [27, 28], while the UTR’s control the translation and half-life of the mRNA. Finally, general production specifics such as HPLC purification and incorporation of modified nucleosides, including 1-methylpseudouridine (m1ψ), make the mRNA non-immunogenic and significantly increase the translation efficiency [29–31].

IVT mRNA is produced from a linear DNA template containing a bacteriophage promotor, the optimized UTR’s and the codon optimized sequence by using a RNA polymerase (T7, T3 or SP6) and a mix of the different nucleosides [7]. The cap structure and the poly A tail can be incorporated during transcription but can also be added enzymatically after the IVT. The resulting product is extensively purified to get rid of contaminants like short transcripts or dsRNA [32, 33]. In this way the IVT mRNA resembles fully processed mature mRNA as it occurs naturally in the cytoplasm of eukaryotic cells.

The series of modifications to the vector used to produce mRNA as well as to the synthetic mRNA itself has ameliorated the biological properties of IVT mRNA. These improvements led to the entry of mRNA therapeutics in different application fields. The first field of entry was the therapeutic cancer vaccination field [34–38], in which a lower safety bar is needed. As the IVT mRNA gets further optimized leading to further reduction of the inflammatory side effects, IVT mRNA entered a second field of application, namely the field of prophylactic vaccination [39, 40]. There are different clinical studies passed and ongoing for IVT mRNA in the cancer vaccination as well as in the prophylactic vaccination field. Next to this two fields, there is an interest in IVT mRNA as protein replacement therapy [19, 31]. However, this is highly challenging as it requires targeted expression of the mRNA and repeated administration, in some cases even a systemic delivery. These requirements imply a high safety bar, making it very challenging. So far no clinical studies are initiated with IVT mRNA in the protein replacement field. Only recently, mRNA also entered the field of gene editing to transiently express the required enzymes inside cells. For example, there is a move to transiently express zinc finger nucleases (ZFNs) using mRNA based vectors as ZFNs can have off-target effects at non-targeted chromosomal sites that are similar in sequence to the intended target site [41–43]. Likewise, mRNA encoding Transcription Activator-like Effector Nucleases (TALENs) is on the market [44–46]. But the newest tool in the genome editing world is the clustered regularly interspaced short palindromic repeats (CRISPR)/CRISPR-associated (Cas) system. Also for this system mRNA encoding the Cas cutting protein can be used [47].

**Fig. 3:**
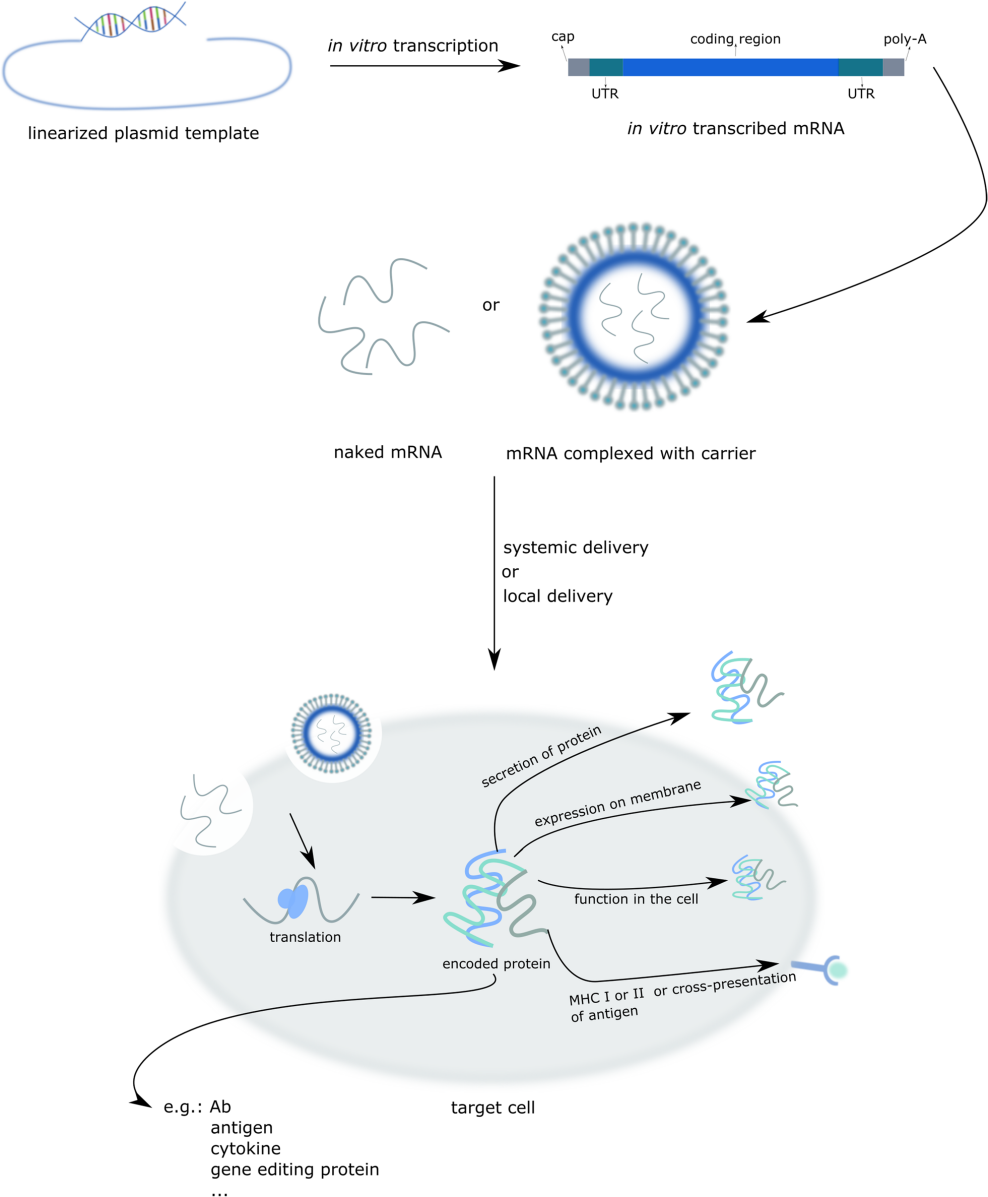
An overview of IVT mRNA based therapeutics. In vitro transcription is performed on a linearized DNA plasmid template containing the coding sequence of interest. Naked mRNA or mRNA complexed in a particle can be delivered systemically or locally. Subsequently, a part of the exogenous naked mRNA or complexed mRNA is taken up by cell-specific mechanisms. Once in the cytoplasm, the IVT mRNA is translated by the protein synthesis machinery of the host cell, after which the protein, depending on its design, can exert its function or be processed as intended

Figure 3 gives an overview of mRNA based therapeutics.

### The genesis of mRNA as platform for mAb production

The first allusion of the concept of using mRNA encoding for antibodies instead of mAb proteins was in 2008 by Hoerr et al. in a patent application under the name RNA-coded antibody (EP 2101823 B1), with CureVac AG as applicant. In March 2017 the first peer-reviewed publication showing the feasibility of using mRNA for passive vaccination was published by Pardi et al. [48]. In this publication, m1ψ-containing mRNAs encoding the light and heavy chains of VRC01, a broadly neutralizing antibody against HIV-1 [49], were formulated in lipid nanoparticles (LNP) and delivered systemically. Pardi et al. already showed that the intravenous injection of mRNA-LNP leads to a robust protein expression in the liver [50]. After a single intravenous injection of 30 µg mRNA-LNP encoding the VRC01 Ab, VRC01 serum levels peaked 24 h after injection followed by gradual decrease until day 11 post injection. The therapeutic capacity of the mRNA-LNPs encoding VRC01 was shown in a prophylactic mice model for HIV-1 and outperformed the recombinant purified protein VRC01 mAb delivery [48].

The feasibility of using mRNA for passive vaccination was independently confirmed a few months later by Thran et al. in three different disease models: as anti-pathogen therapy (rabies model), as an anti-toxin therapy (botulism model) and as an anti-tumor therapy (lymphoma model) [51]. They showed that a single injection of mRNA-LNP encoding either mAbs or camelid heavy-chain only antibodies (VHHs) is sufficient to establish a rapid, strong and long-lasting serum antibody titer in vivo. These high titers lead to full protection against virus challenge or intoxication and could eradicate neoplastic cells in murine models. In addition, the general tolerability of the mRNA-LNP treatment was investigated. Although a transient low increase of certain cytokines was detectable in circulation, this weak increase did not hamper high protein expression. More importantly, histopathology of the liver, the target organ of mRNA-LNPs, did not reveal any sign of abnormality or inflammation.

In the same period a third player entered the field of mRNA encoding antibodies. Stadler et al. reported preclinical data for a new class of drugs that instruct the body to create its own bispecific antibodies, called RiboMABs, a form of bispecific T cell engaging antibodies or BiTEs. These act by connecting human immune cells to tumor cells for highly efficient killing and have demonstrated great promise as immunotherapy agents. A successful example in the clinic is blinatumomab [52], used to treat acute lymphoblastic leukemia [53, 54], a bispecific antibody targeting CD19 and CD3ε aimed at clustering T cells to lymphoma cells [55]. Irrespective of their great potential, most bispecific antibody formats suffer from demanding procedures of production, purification and formulation of the recombinant protein. In addition, the low serum half-life of bispecific antibodies (less than 2 h in patients), warrants a continuous infusion for therapy [56], hindering the development of new drugs in this class of therapeutics. To circumvent these limitations, Stadler et al. engineered IVT modified mRNA encoding bispecific Abs, termed RiboMABs, directed against the T cell receptor associated molecule CD3 and one of three tumor associated antigens (TAA). RiboMABs should be easier to administer and require less frequent dosing than a conventional protein based bispecific Ab. But the major advantage is on the development side as you can easily change the DNA, make the RNA from it, and compare it to other candidate antibody constructs. This fast procedure allows to evaluate different antibodies in a very short period of time.

A few micrograms of the mRNA encoding RiboMAB was formulated into nanoparticles and injected intravenous to get rapid antibody production in liver cells and secretion into circulation. Levels of RiboMAB in serum peaked within hours after injection and remained there for a week at therapeutically effective concentrations. Next, the RiboMABs were tested in xenografts mice models with large ovarian tumors. A 3 weekly treatment with RiboMAB completely eliminated the cancer, comparable with the effectiveness of the corresponding recombinant bispecific antibody, albeit the latter had to be administered three times as often to achieve the same degree of tumor eradication.

The above discussed studies deliver the mRNA intravenously, entailing that the liver is used as bioreactor to translate the mRNA and provide the antibody systemically. In contrast, Tiwari et al. reported on the local delivery of mRNA encoding for both anti-respiratory syncytial virus (RSV) mAb (palivizumab) and VHH [57]. As protection from an infection with RSV requires protective antibodies only in the lungs instead of the whole body, a local delivery of the mRNA encoding Ab is more desirable. The authors used naked mRNA encoding for both a secreted and a membrane-anchored form of Palivizumab or an anti-RSV VHH that was delivered to the lungs via intratracheal aerosols. They were able to show that using this delivery method up to 45% of the lung cells showed detectable antibody expression, leading to a strongly reduced infection 4 days post RSV challenge in the case of secreted Ab and even 7 days in the case of anchored VHH. Importantly, Tiwari et al. showed that upon delivery of naked mRNA via intratracheal aerosols no significantly elevated cytokine levels were detected in the lungs 24 h after treatment. Table 1 gives an overview of pre-clinical studies on mRNA encoding Ab.

**Table 1:** Overview of pre-clinical studies on mRNA encoding Ab

Application field	Ab-format	Pre-clinical study
*Oncology*		
Lymphoblastic leukemia	BiTE	Stadler et al. [94]
Non-hodgkin lymphoma	mAb	Thran et al. [51]
*Infectious disease*		
HIV	mAb	Pardi et al. [48]
HIV, influenza B, rabies	mAb	Thran et al. [51]
RSV	mAb, VHH	Tiwari et al. [57]
*Toxins*		
Botulism	VNA/VHH	Thran et al. [51]

### The unmet needs for mRNA to become a successful therapeutic

#### Activation of the immune system by IVT mRNA

The immunogenicity of IVT mRNA is the primary issue hampering the development of mRNA as a pharmaceutical. Eukaryotic cells express different pattern recognition receptors (PRRs) to recognize structures that are hallmarks of infections. mRNA is recognized by PRRs, such as Toll-like receptor (TLR) 3, 7, 8 and retinoic acid-inducible gene I (RIG-I), resulting in the expression of pro-inflammatory cytokines or inflammasome activation. TLR3 and TLR7/8 respond to double-stranded and single stranded RNA, respectively. The systemic delivery of conventional and unpurified IVT mRNAs can activate the immune system and subsequently leads to the production of pro-inflammatory cytokines and type I interferons [58–61].

Foreign RNA has (potentially) a different pattern of base modifications compared to endogenous RNA. The incorporation of naturally occurring modified nucleosides can (partially) circumvent the recognition of the mRNA by PRR, and in this way decrease immunostimulation and enhance the expression the encoded protein [29, 62–64]. For example, TLR 7 and 8 activation can be avoided by incorporating naturally modified nucleosides such as pseudouridine, 2-thiouridine, 5-methylpyridine, *N*^1^-methylpseudouridine or 5-methylcytidine. Moreover, pseudouridine and 2-thiouridine were shown to make IVT mRNA undetectable by RIG-I and PKR [30, 63]. Previous studies using IVT mRNA in which all uridines were exchanged for pseudouridines, the most common naturally occurring modified nucleoside, showed that the mRNA was nonimmunogenic [29]. Next, Kormann et al. showed that the combination of chemical modifications, 2-thiouridine and 5-methylcytosine, reduced recognition of the mRNA through pattern recognition receptors, including TLR3, 7 and 8 and RIG-I in human PBMCs. Recently, it has been shown that the incorporation of *N*^1^-methylpseudouridine (m1ψ) in mRNA resulted in innate immune evasion and increased translational capacity in vitro and in vivo [65]. In short, the chemical modification of nucleosides in mRNA is an important technology to regulate the immunogenicity of mRNA and is subject of ongoing research.

Unfortunately, modified nucleoside-containing RNA transcribed by phage RNA polymerase transcription still retains a low level of activation of innate immune response pathways [19, 29, 66]. The remaining activation of RNA sensors by nucleoside modified RNA could be because the modifications do not completely suppress the RNA’s ability to activate sensors or due to contaminants with structures that activate in the presence of nucleoside modification. It is well established that RNA transcribed in vitro by phage polymerase contains multiple contaminants, including short RNAs produced by abortive initiation events and double stranded RNAs generated by self-complementary 3′ extensions, RNA-primed transcription from RNA templates and RNA-dependent RNA polymerase activity [67, 68]. For example, dsRNA activates RIG-I, MDA5, PKR and the 2′–5′ oligoadenylate synthetase [69–71]. High-performance liquid chromatography (HPLC) purification removes dsRNA and other contaminants from in vitro transcribed RNAs, yielding higher translation with no release of type I IFNs or TNF-α and no significant induction of genes associated with RNA sensor activation [31].

Next to this, the receptors RIG-I, IFIT1 and MDA are able to discriminate different cap structures [72–74]. Decades of research have established that the m7G cap serves as a unique molecular module that recruits cellular proteins and mediates cap-related biological functions such as pre-mRNA processing, nuclear export and cap-dependent protein synthesis. Only recently, the role of the cap 2′ *O*-methylation as an identifier of self RNA, distinguishing it from foreign RNA and aiding in the innate immune response to foreign RNA, has become clear. These new findings underscore the importance of a proper cap structure in the synthesis of functional messenger RNA and supports the search to better cap-structures. mRNA can also be treated with phosphatase to remove uncapped 3′ triphosphate ends.

Despite the above described adaptations to the IVT mRNA, the emergence of ADA (anti-drug antibody) responses and transient cytokines is still detectable and therefore hampering the clinical applicability of mRNA-drugs, especially when the mRNA has to be administered multiple times.

However, the intrinsic immunogenicity of mRNA can be seen as a double-edged sword. On the one hand, the (systemic) delivery of conventional and unpurified IVT mRNAs can activate the immune system and subsequently lead to the unwanted (systemic) production of pro-inflammatory cytokines and type I interferons (IFNs) [58–61]. This intrinsic immune-stimulatory activity can directly interfere with the aimed therapeutic outcome, for example in the case of gen replacement therapy, as it can reduce the expression of the encoded protein. On the other hand, in certain application fields such as vaccination approaches, the inflammatory cytokine production resulting from the recognition of the mRNA might add to the effectiveness of the evoked immune response, making the mRNA to become its own adjuvant. The intrinsic adjuvant properties of mRNA appears to be mainly based on its capacity to evoke type I IFNs [75]. The impact of type I IFNs on T cell immunity can be either beneficial or detrimental, depending on their kinetics of induction, intensity and anatomical distribution. This way, Type I IFNs exert profound stimulatory effect upon intravenous injection [38, 76] but potent inhibitory effects upon topical injection [77]. Next to these concerns, the in vivo application of mRNA has been confronted with considerable skepticism as mRNA is believed to have a short extracellular half-life because of the omnipresent RNases.

#### Delivery of mRNA

The development of mRNA therapeutics faces the same challenge as any nucleic acid, namely the issue of delivery. As a negatively charged, high molecular weight molecule, mRNA is intrinsically unsuited to cross cellular membranes and to reach its target location, the cellular cytosol. The difficulties in achieving efficient delivery have seriously hampered the application of RNA for drug development. For this reason, a variety of approaches have been evaluated, including optimized injection strategies, gene gun-based administration, protamine condensation, RNA adjuvants and encapsulation of RNA in nanoparticles consisting of polymers and liposomes [78]. In theory, exogenous RNA needs to cross one lipid bilayer to become internalized by target cells and translated into a functional antigen. Naked mRNA is spontaneously taken up by many different cell types [79–81]. Most cell types do internalize mRNA through various internalization pathways, resulting in mRNA to become entrapped and degraded in acidic endolysosomal compartments. Dendritic cells (DCs)—immune cells specialized in antigen presentation and nucleic acid sensing—appear to constitute an exception to this rule, as they have been reported to express naked mRNA with reasonable efficiency upon intranodal or intratumoral administration. How DCs shuttle mRNA to the cytosol remains largely unknown, but it appears to involve a macropinosome to cytosol shuttling mechanism, similar as been described for protein antigens internalized by DCs. It has been shown that the uptake of naked RNA by immature DCs is an active process, which involves scavenger receptor-mediated endocytosis and micropinocytosis [79–81]. Both pathways lead to endolysosomal localization, whereafter only a small fraction of intact RNA enter the cytoplasm [81]. To deal with this, multiple formats have been designed to both target the mRNA to antigen presenting cells as well as to augment the amount of RNA reaching the cytosol after uptake. Mostly all developed approaches are based on nanoparticle formation, such as the use of liposomes, polymers and peptides.

Not all cells produce an equal level of protein after i.v. administration of mRNA lipid nanoparticles. This depends on the type of ionizable lipid and the formulation of the lipid (PEG-lipid) as described in the paper of Paunovska et al. [82]. Next to this, the UTR sequences determine the degree of tissue and cell specificity as described in the paper of Jain et al. [83]. Fenton et al. recently described lipid nanoparticles that selectively target B cells in the spleen [84].

Nonetheless, efficient mRNA expression in most cell types does require the mRNA to be encapsulated into nanoparticulate delivery vehicles that aid cellular uptake and mediate mRNA escape from endosomes to cytosol. To complex negatively charged mRNA, cationic lipids are perfectly suited as both components spontaneously interact to form lipoplexes [85]. Lipoplex-based delivery of mRNA has two main benefits. First, the mRNA is condensed into particles within the range of micro-organisms, resulting in efficient targeting and uptake by professional APCs. Second, in a condensed state, the mRNA is less vulnerable for intracellular and extracellular enzyme-mediated degradation. To develop safe and powerful delivery vehicles suited for mRNA delivery, the mRNA field is currently strongly capitalizing on the vast knowledge gained during the clinical development of small interfering RNAs (siRNA). Efficient systemic delivery of siRNA can be achieved by its encapsulation into so called LNPs, with the first RNAi LNP drug product (Patisiran) been approved by the FDA August 2018 and several others in advanced clinical studies. Lipid based nanoparticles are composed of four different lipids with specialized functions, which are mixed at variable ratios with RNA under acidic conditions. Ionizable lipids or lipid-like materials constitute one of the most critical components of these LNPs and are responsible for mRNA complexation by charge interaction and for endosomal release of mRNA. Compared to earlier lipids bearing a permanent cationic charge, these new generation lipids contain amine groups that are cationic at acidic pH but neutral at physiological pH, which reduces toxicity and improves efficiency. Besides the ionizable lipid, LNPs typically contain cholesterol, a helper lipid and a PEGylated lipid.

Although LNPs are promising delivery vehicles, significant hurdles in terms of safety need to be addressed to enable clinical development of mRNA LNPs. To date, clinical data on the safety and efficacy of mRNA LNPs are scarce, and ongoing phase studies are still limited to topical (intramuscular, intratumoral) administration of LNP packaged mRNA. Following encouraging results in rodents and non-human primates, Moderna initiated a first-in-human study in which the immunogenicity and safety of a low dose (100 µg) mRNA LNP vaccine encoding influenza HA was assessed (clinical trial NCT03076385). Interim findings were reported in early 2017 and demonstrated that the vaccine induced sufficient immunogenicity and an acceptable tolerability profile. Nonetheless, even at this low dose, mild to moderate reactogenicity was encountered in most and 3/23 subjects displayed either high local reactogenicity or systemic side effects. Although these data support further development of mRNA LNP therapeutics, they also suggest safety hurdles still need to be taken to enable clinical development, especially if repeated systemic administration of high doses (mg/kg) of mRNA LNPs is envisioned, as is likely in case of mRNA encoded antibodies.

Toxicity of mRNA LNPs can be multifaceted, including immune related toxicity events as well as cellular toxicities caused by the accumulation of lipids in the liver. Similar to other nanomedicines, LNPs have been reported to activate the complement system, which harbors the risk of eliciting a hypersensitivity reaction known as complement activation related pseudoallergy (CARPA) [86, 87]. In addition, for stability reasons, LNPs contain polyethyleneglycol modified lipids that can activate splenic B cells to produce anti-PEG antibodies. Such anti-PEG antibodies not only have been associated with antibody mediated anaphylactic responses upon secondary exposure, but also underlie the so-called accelerated blood clearance effect (ABC), by which opsonized LNPs are rapidly cleared by macrophages, and which results in gradually decreasing mRNA expression levels upon each sequent administration. The extent at which these anti-PEG antibodies are induced is governed by two factors: (i) the immunogenicity of the mRNA and (ii) the fatty acid chain length of the PEG-lipid used. As explained above, non-purified, non-modified mRNA is recognized by various innate RNA sensors, resulting in the secretion of inflammatory cytokines that promote the differentiation of PEG-recognizing B cells into antibody secreting plasma cells. Highly pure, innate silent mRNA avoids these adjuvant effects and hence restricts B cell activation. A second major determinant of PEG immunogenicity is the chain length of the fatty acid chains of the PEG-lipid used to stabilize the LNPs. The Cullis’ group nicely demonstrated that C18 PEG-lipids (e.g. distearolyglycerol) are very stably incorporated into the LNPs, whereas C14 PEG-lipids rapidly dissociate from the LNP in blood [88–90]. This highly stable incorporation of C18 PEG-lipids into LNPs extends and intensifies the exposure of splenic B cells to the PEG moieties, which translates in strongly increased anti-PEG antibody titers for C18 PEG LNPs. Combining innate silent mRNA with C14 PEG based LNPs appears to be an extremely successful approach to avoid hypersensitivity reactions and the ABC effects, as exemplified by recent preclinical studies, at least at relatively low doses and in rodents.

## The specific challenges and perspectives of mRNA as antibody platform

Until now, only a few pre-clinical studies, mostly in small rodents, have been performed for mRNA encoding Abs. Hence, the expression and efficacy still has to be demonstrated in larger animals and ultimately in humans. That being said, an earlier study on another type of secreted protein, namely erythropoietin, revealed that findings in a murine model can be translated to larger animals such as domestic pigs or even primates [91]. These results give hope that the data on mRNA-encoded antibodies in murine models are potentially translatable to humans as well.

A pharmaceutically applicable mAb has to comply to three rules: (i) there needs to be a fast increase of serum titers after injection, (ii) the antibody serum titers have to be high enough to give protection and (iii) the half-life of serum titers has to be long enough.

The increase and level of mAbs are determined among other things by the formulation and route of delivery. Mostly all developed approaches to deliver mRNA are based on nanoparticle formation, such as the use of liposomes, polymers and peptides. Pardi et al. showed that mRNA packaged in lipid nanoparticles enables high levels of protein production for an extended period of time when administered by a variety of routes [50]. So far, only lipid nanoparticle based delivery has been used for mRNA encoding Ab.

Next to the formulation of the mRNA, also the delivery route has an impact on the induced serum titers of the Ab. There are three types of delivery routes investigated so far for mRNA vaccines in general: local delivery [57, 92, 93] (e.g. intrapulmonary, intradermal and subcutaneous), targeted delivery [34] (intranodal) or systemic delivery [38, 76] (intravenous). Recent literature shows that mRNA encoding Abs leads to antibody titers already detectable the first day after intravenous injection [48, 51, 94]. So far, only the study of Tiwari et al. showed data on local delivery to the lungs through intratracheal aerosols [57]. Next to these two delivery methods, no other routes of administration have been tested for mRNA encoding Abs. This is important in the light of approved therapies, as the targeted delivery of the mRNA to the organ of interest has the potential to minimize systemic toxicity, anti-antibody immune responses and reduce the amount of drug required to achieve therapeutic levels. But the targeted delivery may also reduce the number of target cells reached by the mRNA and as a consequence reduce the cells that produce the encoded Ab [95].

Therapeutic doses of recombinant antibodies used today are often quite high. So far it is not clear if these high doses are achievable by administration of encoding mRNA. Nonetheless, several considerations argue in favor of mRNA over recombinant antibodies. Firstly, it is possible that in situ antibody expression may reduce the amount of protein needed for therapeutic effect because of the high local concentration. Secondly, based on the doses tested so far, no saturation nor dose-limiting toxicity has been detected for mRNA mediated antibody delivery. Thirdly, it is likely that target-specific mRNA optimizations and further improvements to the formulation can substantially increase efficacy [91].

The serum half-life of Ab encoded by mRNA is determined by the half-life of the Ab itself on the one hand and the mRNA encoding the Ab on the other hand. More specifically, the half-life during the first phase is determined by the mRNA and protein half-life, while in the second phase the half-life is almost exclusively determined by the protein properties. This entails the half-life of short-lived proteins can significantly benefit from being expressed by mRNA [62, 91]. In case of long-lived proteins, the use of an mRNA expression platform has no apparent impact on the duration of the therapeutic effect, but mRNA half-life does contribute to peak levels expression. Also the size of the antibody molecule restricts its applicability in mRNA form. For example, the commonly used IgG isotype, is in the range of 150 kDa. Next to this, antibodies are complex multidomain proteins that have to assemble in a correct way. To overcome the size and stability limitations of mRNA-encoded mAb, a lot of research has been done on both heavy-chain only antibodies or VHH and the generation of small non-antibody based scaffolds [96–98]. There are two types of non-antibody scaffolds, namely (i) domain-sized compounds (6 to 20 kDa) like DARPins [99] and alphabodies [100] and (ii) constrained peptides (2–4 kDa) [100]. Different scaffolds are currently under academic, preclinical and clinical development and have shown great potentials in terms of affinity, target neutralization and stability [97, 101, 102]. Nonetheless, non-antibody based scaffolds face their own challenges, of which serum-half life and tissue penetration are the most important ones. It is of particular interest to investigate the efficacy of mRNA encoding for non-antibody based scaffolds as the mRNA platform can have a positive effect on the serum half-life as discussed above.

## Conclusions

The industry of mAb therapeutics is one of the fastest growing segments in the pharmaceutical world with applications in different fields. A great body of knowledge has been accumulated on the production and use of mAb as protein. Astonishing is the fact that only a few of the more than 70 mAbs that have been licensed, are directed against infectious diseases, for example Obiltoxaximab and Raxibacumab against anthrax infection and Palivizumab against respiratory syncytial virus. This is stunning as in cases where antibiotics fail or antivirals are not available, mAbs represent a powerful alternative to combat infectious diseases [103–105]. Reasons for this can be found in the money- and time-consuming production and purification processes, extensive downstream quality control and the need for a cold supply chain [106, 107]. The higher costs related to protein based mAbs are in sheer contrast with the costs for most small molecule drugs or antibiotics.

To circumvent the problems of complex production and purification processes and aberrant posttranslational modifications of protein based mAb, alternative ways are now being explored to rapidly produce, administer and test mAb. Nucleic acid therapeutics have great potential as they are simple, fast and cost effective as it does not require complex and expensive laboratory infrastructures, with a generic production process for all mRNAs [7, 8]. Recent advances with mRNA, including improvements with in vitro transcription, have increased interest in the therapeutic potential of this biomolecule. Unlike DNA, mRNA only needs to reach the cytoplasm to induce protein expression and, additionally, bears no apparent risk of insertional mutagenesis. The mRNA based platform for Ab therapeutics has different advantages over the protein based platform. First of all, with mRNA expression of the encoded Ab is detectable for a few days in contrast to a single protein shot with the protein-format. Secondly, with mRNA it is easier to deliver intrabodies as mRNA is transfected in the cells while it is much harder to get the protein-format in the cells. And thirdly, as proteins are composed of 20 different amino-acids as building blocks, the physicochemical properties differ from protein to protein, implying that for each protein the buffer for storage and formulation should be optimized singly. mRNA on the other hand uses a mere four nucleosides as building blocks, leading to a structure that has largely consistent physicochemical characteristics regardless of the protein sequence it encodes.

While the first peer-reviewed studies with mRNA-based antibodies were only recently published [48, 51, 57, 94], this application has matured behind corporate walls for decades. So far the applicability of the mRNA platform for antibody therapy is investigated in the context of antitoxins, infectious diseases and oncology. Notwithstanding that the first reports indicate that mRNA presents an emerging platform for antibody gene transfer, the further development of mRNA based mAb is limited by the need for safe and effective delivery systems. Next to this, mRNA can only lead to Ab with natural post-translational modifications, meaning that conjugates and modifications to increase serum half life (for example by PEGylation) are not possible for Ab encoded by mRNA.

For passive immunization a very high safety profile is required. Over the past decades, different optimisations are described for IVT mRNA in order to avoid unwanted immune activation and cytokine induction induced by cellular RNA sensors. Despite the above described adaptations to the IVT mRNA, the emergence of ADA (anti-drug antibody) responses and transient cytokines is still detectable and therefore hampering the clinical applicability of mRNA-drugs, especially when the mRNA has to be administered multiple times. Notably is also the induction of CARPA when nanoparticles are given repeatedly. This formulation has to be analyzed more carefully as mice are barely sensitive to CARPA induction.

In conclusion, mRNA encoding Abs are a viable therapeutic option that can circumvent the problems of complex production and purification processes and aberrant posttranslational modifications inherent to protein based mAb. Nonetheless, to successfully replace the current protein antibody format, mRNA approaches will need to surpass their own challenges, which are mainly situated at the level of delivery and immunogenicity. But with the emergence of mRNA as a therapeutic and the growing research on this topic, mRNA therapeutics will likely further evolve and improve the coming years.

## Abbreviations

m1ψ: 1-methylpseudouridine
ABC: accelerated blood clearance effect
ATMPs: advanced therapy medicinal products
ADA: anti-drug antibody
ADA: anti-drug antibody
APCs: antigen presenting cells
BiTEe: bi-specific T cell engagers
VHHs: camelid heavy-chain only antibodies
CRISPR: clustered regularly interspaced short palindromic repeats
CARPA: complement activation related pseudoallergy
CAS: CRISPR-associated
DC: dendritic cell
EMA: European medicines agency
FDA: food and drug administration
GTMP: gene therapy medicinal product
HPLC: high-performance liquid chromatography
HIV: human immunodeficiency virus
IVT: in vitro transcribed
IFNs: interferons
IM: intramuscular injection
LNP: lipid nanoparticle
mRNA: messenger ribonucleic acid
mAbs: monoclonal antibodies
PRRs: pattern recognition receptors
pDNA: plasmid DNA
RSV: respiratory syncytial virus
RIG-I: retinoic acid-inducible gene I
siRNA: small interfering RNAs
TLR: toll-like receptor
TALEN: transcription activator-like effector nuclease
TAA: tumor associated antigens
VNA: VHH based neutralizing agent
ZFNs: zinc finger nucleases
